# The Ministry of Pensions. Prompt Reorganisation Essential

**Published:** 1919-01-04

**Authors:** 


### THE MINISTRY OF PENSIONS.
### PROMPT REORGANISATION ESSENTIAL.

The medical profession has a vital interest in the placing of the Ministry of Pensions under the supreme direction of the ablest administrator whose services can be made available for this important office. It would be a great injustice, and no small evil, to the wounded and physically disabled or permanently affected warrior, to delay longer placing the direction of the heavy and most responsible work at the Ministry of Pensions, in the hands of an organisation controlled and directed by a man of great experience, technical knowledge and approved ability. It is essential to create the requisite means, and to provide for their prompt and continuous application, so as to secure the maximum of efficiency, and the minimum of delay and trouble to each permanently injured and sick warrior when he becomes largely dependent for his comfort and safety upon the perfect administration of the Ministry of Pensions. The vastness of the work, its complications and difficulties, and the necessity of averting danger from infection to the civil population combine to render it essential for the Prime Minister to give immediate and particular attention to the Ministry of Pensions. Mr. Lloyd George has been fortunate in the election of his lieutenants, and much must depend upon his selection of a man or men whose record and service will be a guarantee to the nation that the Ministry of Pensions has been placed by the Prime Minister in adequate hands. Then the whole machinery involved, and the help to be afforded should prove ample to make them speedily ready and continuously efficient to secure the maximum of benefit to all dependent on this department. As a nation, though we possess a number of administrators of the first rank, they are relatively few in number, and to secure a wise selection it is essential that the Prime Minister, in arranging for the higher appointments to the Ministry of Pensions, should make it his business to pass by types of men who, however enthusiastic and philanthropic they may be in their attitude towards the wounded, are without the essential experience and technical qualifications, so pressingly needed to convert the Ministry of Pensions into a most efficiently administered and continuously successful department of the State.

Our wounded and disabled warriors are properly regarded by all sections of the public as children of the State. They must have complete justice and the most generous consideration and tender handling, so that nothing may be left undone to expedite the provision for every one of them of suitable accommodation, and the speediest settlement of their claims and needs. It is an open secret, which has been the subject of more than one article in the *Times*, that the Ministry of Pensions requires reorganisation, involving certain changes in the personnel, including the selection of the best and most experienced administrator the Prime Minister can put his hands on. Any delay must mean avoidable suffering, disappointment and possibly hardship to the returning warriors whose future must necessarily and largely be in the hands of the Ministry of Pensions. It was essentially difficult to commence this Ministry at the outset, and the difficulties have not decreased with time. Less than three weeks ago a decision was announced to the effect that the Army Medical Service should treat all pensioners and disabled soldiers for a period of six months from December 3. The medical work of the Ministry of Pensions is rightly regarded by the War Office authorities as of vital and far-reaching importance. It entails for its success that the medical departments of the Army and the Ministry of Pensions work in the closest possible harmony and co-operation to secure the welfare of the disabled soldier. The Ministry of Pensions requires reorganisation and its successful working should be secured and perfected at the earliest possible moment. It would be a sin to permit inefficiency to become rampant at the Ministry of Pensions. The absence of adequate administrative control cannot longer be suffered to hamper the fate of our injured warriors, their maximum of skilful treatment, and their continuous welfare.

